# Comparison of the Swedish and Finnish Centers in the European Randomized Study of Screening for Prostate Cancer (ERSPC)

**DOI:** 10.1016/j.euros.2026.04.008

**Published:** 2026-05-15

**Authors:** Marianne Månsson, Kirsi Talala, Anssi Auvinen, Jonas Hugosson

**Affiliations:** a Department of Urology, Sahlgrenska Academy at University of Gothenburg, Gothenburg, Sweden; b Cancer Society of Finland, Helsinki, Finland; c Prostate Cancer Research Center, Faculty of Social Sciences, Tampere University, Tampere, Finland

**Keywords:** Cancer-specific mortality, Contamination, Prostate cancer, Prostate-specific antigen, Screening

## Abstract

**Background and objective:**

The European Randomized Study of Screening for Prostate Cancer showed 20% reduction in prostate cancer (PC) mortality after 16 year follow-up of prostate-specific antigen (PSA) based screening, but effects varied: 9% in Finland (FinRSPC) vs 37% in Sweden (Göteborg-1) in screening vs control groups. The objective was to explore this difference.

**Methods:**

The data comprised 64 774 participants in FinRSPC and 10 703 men in Göteborg-1, aged 55–63 year at randomization (FinRSPC: 1996–1999; Göteborg-1: 1995). Screening invitations were issued for PSA testing every 4 year, with biopsy recommended if PSA ≥4 ng/ml, or 3–3.99 ng/ml with an additional test in FinRSPC; and every 2 year, with biopsy recommended at PSA ≥3 ng/ml in Göteborg-1. Expected incidence/mortality was based on registry data (1990–1994, and 2010–2014). Outcomes included cumulative PC incidence, PC-specific mortality, and relative risks.

**Key findings and limitations:**

In Göteborg-1, PC incidence was 44% higher (95% CI [confidence interval]: 29–61) and PC mortality 36% lower (95% CI: 7–56) in screening vs control groups. In FinRSPC, PC incidence was 21% higher (95% CI: 15–27) and PC mortality 2% lower (95% CI: –19–19). Screening groups in both centers had approximately 40% lower mortality than expected with 1990–1994 rates, similar to 2010–2014 rates. Swedish controls resembled expected rates in 1990–1994 and Finnish controls in 2010–2014. A key limitation was the exploratory nature of the study.

**Conclusions and clinical implications:**

The lower effect on PC mortality in FinRSPC appears attributable to unexpectedly low mortality in the control group, possibly due to extensive contamination from the start of the study. Findings highlight the challenges in demonstrating mortality benefit in screening trials under widespread opportunistic testing.

## Take Home Message

The Finnish and Swedish parts of the European Randomized Study of Screening for Prostate Cancer trial reported marked difference in prostate cancer mortality effects after 16 year. Extensive contamination of the Finnish control group from the study’s start may explain the smaller mortality reduction in FinRSPC.

ADVANCING PRACTICE **What does this study add?**

FinRSPC and Göteborg-1 are two prostate cancer screening trials that, after 16 years of follow-up, reported markedly different effects on prostate cancer mortality. Using trial data combined with historical registry data, we investigated the reasons for this discrepancy. Our results suggest that the limited mortality reduction in the Finnish trial is likely due to more extensive opportunistic testing in the Finnish control group already from study start, resulting in higher prostate cancer incidence and subsequent lower prostate cancer mortality. These findings suggest that demonstrating a mortality benefit of screening may be challenging in settings with substantial opportunistic PSA testing.

**Clinical Relevance**

The effectiveness of PSA-based prostate cancer screening is highly dependent on the population invited, including their baseline risk and prior exposure to testing. In particular, high rates of ongoing opportunistic PSA testing in the background population can substantially dilute the observed mortality benefit in randomized screening trials. These findings underscore that the impact of organized screening cannot be interpreted without considering the underlying screening intensity in both intervention and control groups. An increasingly important aim of organized prostate cancer screening is not only to improve outcomes, but also to reduce inefficient and unstructured opportunistic testing. Associate Editor: Roderick C.N. van den Bergh, MD PhD.

**Patient Summary**

Previous findings have shown a substantial greater reduction in prostate cancer mortality in the Swedish screening trial than in the Finnish trial. One possible explanation is that more extensive opportunistic testing was conducted in the Finnish control group from the study’s start. This may have led to more cancers being detected at a curable stage and thereby reduced the mortality difference between the control and screening groups in Finland.

## 1 Introduction

In the multicenter European Randomized Study of Screening for Prostate Cancer (ERSPC), repeated prostate-specific antigen (PSA) based screening reduced prostate cancer (PC) mortality by 20% after 16 year of follow-up of men aged 55–69 year at randomization [1]. However, mortality reductions differed considerably across the seven participating centers, ranging from 1% to 37%. That there is a variation is not surprising as the screening algorithms between the centers differed. For instance, men were invited for screening every 4th year at most of the centers, but in Sweden it was every 2nd year. At some centers, the indication for biopsy was a PSA level of ≥3.0 ng/ml, while others used combinations of PSA level, findings at digital rectal examination (DRE), transrectal ultrasonography, and/or free-to-total PSA; and the start year of the study ranged from 1991 to 1998 [2]. Moreover, centers differed in terms of compliance, contamination of the control arm, the underlying PC mortality, and incidence rates.

The impact of various study features on PC mortality in ERSPC has been evaluated by microsimulation, and the most important features were attendance, biopsy compliance, and contamination [3], [4]. The variation in results likely reflects both the underlying risk of PC mortality and protocol differences [5], while a major impact of the method of randomization has been ruled out [6].

Two centers with markedly different PC mortality reductions are Finland (9%) and Sweden (37%) [1]. Two possible explanations for this difference are that either the Swedish screening group experienced a relative advantage compared with the Finnish, or alternatively, the Swedish control group was at a disadvantage relative to the Finnish. The aim of this study was to investigate these possibilities using registry data from periods with very low PSA testing and with widespread opportunistic testing.

## 2 Materials and methods

### 2.1 Study populations

Göteborg Randomized Population-Based Prostate Cancer Screening Trial (Göteborg-1, ISRCTN54449243) and Finnish Randomized Study of Screening for Prostate Cancer (FinRSPC) have been described previously [7], [8], and constitute the Swedish and Finnish sections of the ERSPC trial (ISRCTN49127736).

In Göteborg-1, 20 000 men from the Gothenburg community aged 50–64 year were randomized 1:1 to a screening group (SG) or a control group (CG) on December 31, 1994. Men in the SG were invited to undergo PSA testing every 2nd year. The oldest cohort was invited three times and the youngest ten times. Men with a PSA level of ≥3 ng/ml were invited to a clinical assessment including prostate biopsy, while men with PSA levels below the cut-off level were reinvited after 2 year, as were men with no cancer.

FinRSPC included 80 458 men from the Finnish Population Register between 1996 and 1999. Men aged 55, 59, 63, or 67 year in the Helsinki or Tampere metropolitan areas were eligible. On January 1, each year 8000 men were allocated to the SG, yielding an approximate 1:1.5 ratio between the SG and CG. Men in the SG were invited to screening every 4th year up to three times, except for men aged 67 year at randomization who were invited twice. A PSA level of ≥4.0 ng/ml prompted referral to a local urological clinic for DRE, transrectal ultrasound, and prostate biopsy. Men with PSA levels between 3.0–3.99 ng/ml were referred in case of suspicious DRE findings (1996–1998) or free/total PSA levels of ≤16% (since 1999). Further details are provided in Supplementary Table 1.

Data on vital status and residence were obtained from the Swedish and Finnish population registers, and on PC diagnosis from the Regional Cancer Register of Western Sweden and the Finnish Cancer Registry.

The present study was restricted to men aged between 55 and 63 year at randomization to minimize potential differences in screening effect due to differences in start age. In FinRSPC, there were 25 739 and 39 035 men of this age in the SGs and CGs, respectively, and in Göteborg-1, the corresponding numbers were 5346 and 5357 (after exclusion of 156 and 78 men in FinRSPC and Göteborg-1, respectively, due to death, emigration or PC before randomization). The follow-up was truncated at 16 year from randomization, with censoring at death, emigration, or 16 year, whichever occurred first.

### 2.2 Register data

National data from the Swedish National Cancer Register, Statistics Sweden, the Finnish Cancer Registry, and Statistics Finland were used to compare the PC incidence and mortality observed in Göteborg-1 and FinRSPC to expected outcomes in three cases:

Pre-study: With low (Finland) or negligible (Sweden) rates of opportunistic screening (PC incidence and mortality from 1990–1994).

End-study: With opportunistic testing in the community mirroring that at the end of the follow-up in these studies (2010–2014).

Along-study: With opportunistic testing as it evolved during the study period.

### 2.3 Statistical analysis

Cumulative PC incidence and PC-specific mortality were estimated using the Kaplan-Meier method (1 minus the survival estimate), and expected incidence and mortality based on register data using the Ederer II method [9]. Since prevalent PC cases were excluded in Göteborg-1 and FinRSPC, the observed PC mortality among prevalent cases was subtracted from the expected mortality.

Rate ratios (RR) based on events per person-year between the SG and CG were estimated with 95% confidence intervals (CIs) using the score method [10].

Statistical analyses were performed using R Statistical Software, version 4.5.1.

## 3 Results

The median age at randomization was 59 year, and median follow-up of men still at risk was 16 year in both studies. In Göteborg-1, 76% of invited men attended screening at least once, and 75% in FinRSPC.

The number of PC cases, PC deaths, other-cause deaths, and additional characteristics are presented in Table 1. The RR of PC incidence in the SG versus the CG was 1.44 (95% CI: 1.29–1.61) in Göteborg-1, whereas in FinRSPC the corresponding RR was 1.21 (95% CI: 1.15–1.27). The RR of PC mortality was 0.64 (95% CI: 0.44–0.93) in Göteborg-1 and 0.98 (95% CI: 0.81–1.19) in FinRSPC.

**Table 1:** Characteristics of the randomized men and data on PC diagnosis, PC deaths, and other deaths.

	FinRSPC		Göteborg-1	
	CG	SG	CG	SG
Number of men (*n*)	39 035	25 739	5357	5346
Number of person-years	551 297	362 643	72 587	72 957
Age at randomization, Year	59 [55–63]	59 [55–63]	59 [56–61]	59 [56–61]
Attended at first invitation	–	16 733 (65%)	–	3290 (62%)
Attended at least once	–	19 388 (75%)	–	4089 (76%)
Length of follow-up (excel men with events)	16.0 [15.5–16.0]	16.0 [15.4–16.0]	16.0 [12.9–16.0]	16.0 [13.1–16.0]
Number of PC (per 1000 person-years^a^)	3462 (6.5%)	2694 (7.9%)	521 (7.5%)	723 (10.8%)
Age at PC diagnosis, Year, Median, [IQR]	69.8 [65.7–73.8]	67.7 [63.7–72.3]	70.1 [65.9–74.1]	66.6 [63.4–70.8]
Number of PC deaths (per 1000 person-years)	272 (0.49%)	176 (0.49%)	67 (0.92%)	43 (0.59%)
Number of other deaths (per 1000 person-years)	9777 (18%)	6569 (18%)	1456 (20%)	1457 (20%)

Values are expressed as *n* (%) or median [IQR].

FinRSPC = Finnish Randomized Study of Screening for Prostate Cancer; G1 = Göteborg Randomized Population-Based Prostate Cancer Screening Trial; CG = control group; IQR = interquartile range; PC = prostate cancer; SG = screening group.

a Person-years are here calculated up to the minimum of the dates of PC diagnosis, death or emigration

In the SGs, low-risk was the dominant tumor stage group, representing 60% of all cancers in Göteborg-1 and 43% in FinRSPC (Table 2). In the CGs, the corresponding proportions were 31% and 28%. Low-risk cancer was diagnosed 167% (95% CI: 124–218) more often in the SG than in the CG in Göteborg-1, and 86% (95% CI: 71–102) more often in FinRSPC (Supplementary Table 2). In contrast, high-risk and advanced cancers were diagnosed 37% (95% CI: 21–50) less often in the SG than in the CG in Göteborg-1, and 14% (95% CI 6–21) less often in FinRSPC. In absolute numbers, advanced cancer was diagnosed in 1.5% (CG) vs 0.8% (SG) in Göteborg-1, and 0.9% (CG) vs 0.6% (SG) in FinRSPC.

**Table 2:** Distribution of tumor stage groups among men with PC (upper part of the table) and among men who died from PC (lower part of the table) during 16 year of follow-up.

	FinRSPC		Göteborg-1	
	CG	SG	CG	SG
Distribution of stage group among men with PC				
	*n* = 3462	*n* = 2694	*n* = 521	*n* = 723
Low	955 (28%)	1169 (43%)	163 (31%)	434 (60%)
Moderate	1144 (33%)	716 (27%)	158 (30%)	169 (23%)
High	843 (24%)	526 (20%)	106 (20%)	71 (10%)
Advanced	368 (11%)	162 (6.0%)	78 (15%)	44 (6.1%)
Not known	152 (4.4%)	121 (4.5%)	16 (3.1%)	5 (0.7%)
Distribution of stage group among men who died from PC				
	*n* = 272	*n* = 176	*n* = 67	*n* = 43
Low	12 (4.4%)	15 (8.5%)	1 (1.5%)	2 (4.7%)
Moderate	22 (8.1%)	27 (15%)	7 (10%)	8 (19%)
High	63 (23%)	57 (32%)	13 (19%)	9 (21%)
Advanced	171 (63%)	74 (42%)	44 (66%)	23 (53%)
Not known	4 (1.5%)	3 (1.7%)	2 (3.0%)	1 (2.3%)

Values are expressed as *n* (%).

FinRSPC = Finnish Randomized Study of Screening for Prostate Cancer; Göteborg-1 = Göteborg Randomized Population-Based Prostate Cancer Screening Trial; CG = control group; PC = prostate cancer; SG = screening group.

Definition of tumour stage groups: Low risk: cT1/cT2, GS 6 and PSA < 10; Intermediate risk: (cT1/cT2, PSA 10-20, and GS = 6) or (cT1/cT2, PSA < 20 and GS = 7); High risk: (cT1/cT2, PSA≥20 and GS<=7) or (cT1/cT2 and GS = 8–10) or (cT3); Advanced: N1, M1, cT4 or PSA > 100.

With respect to PC mortality, 66% and 63% of all deaths occurred in men with advanced cancer in the Swedish and Finnish CGs, whereas it was, 54% and 42% in SGs. When high-risk and advanced cancers were combined, approximately 85% and 74% occurred in the CGs and SGs, respectively, in both studies.

**Fig. 1:**
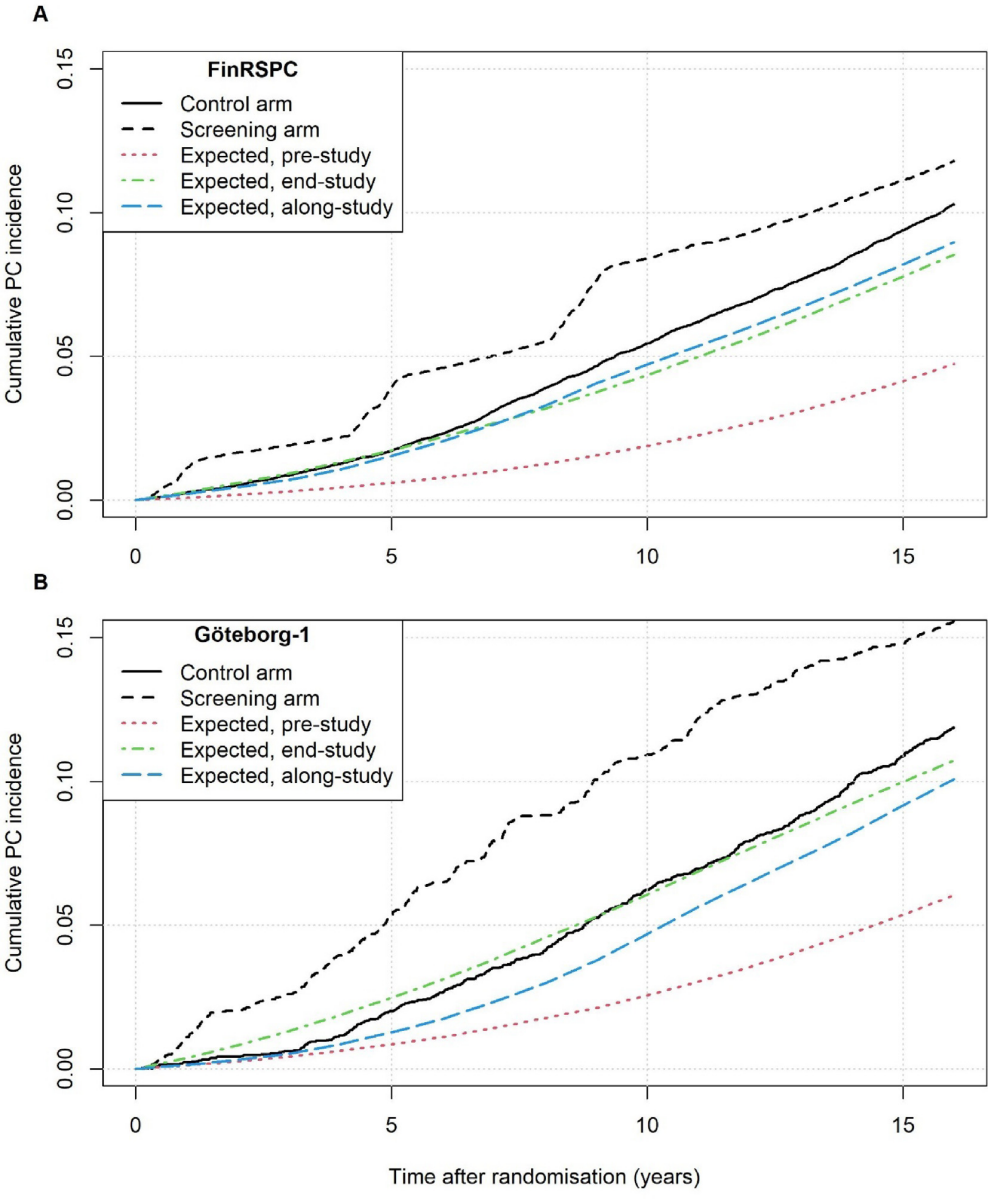
Observed cumulative incidence of PC in the control and screening groups of FinRSPC (A) and Göteborg-1 (B), and estimations of the expected incidence based on population rates from pre-study (1990–1994), along-study, and end-study (2010–2014). PC = prostate cancer.

**Fig. 2:**
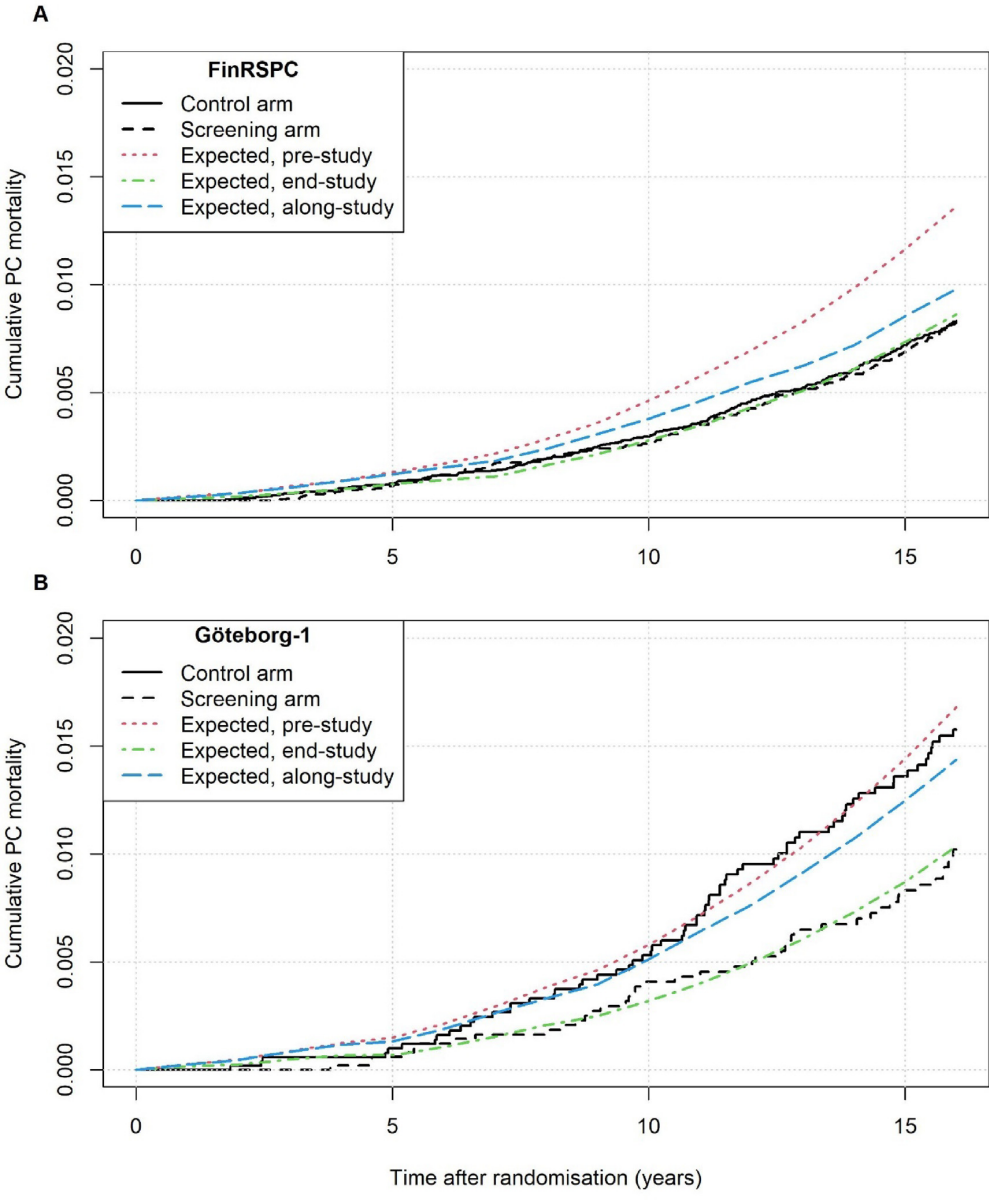
Observed cumulative PC mortality in the control and screening groups of FinRSPC (A) and Göteborg-1 (B), and estimations of the expected mortality based on population rates from pre-study (1990–1994), along-study, and end-study (2010–2014). PC = prostate cancer.

**Fig. 3:**
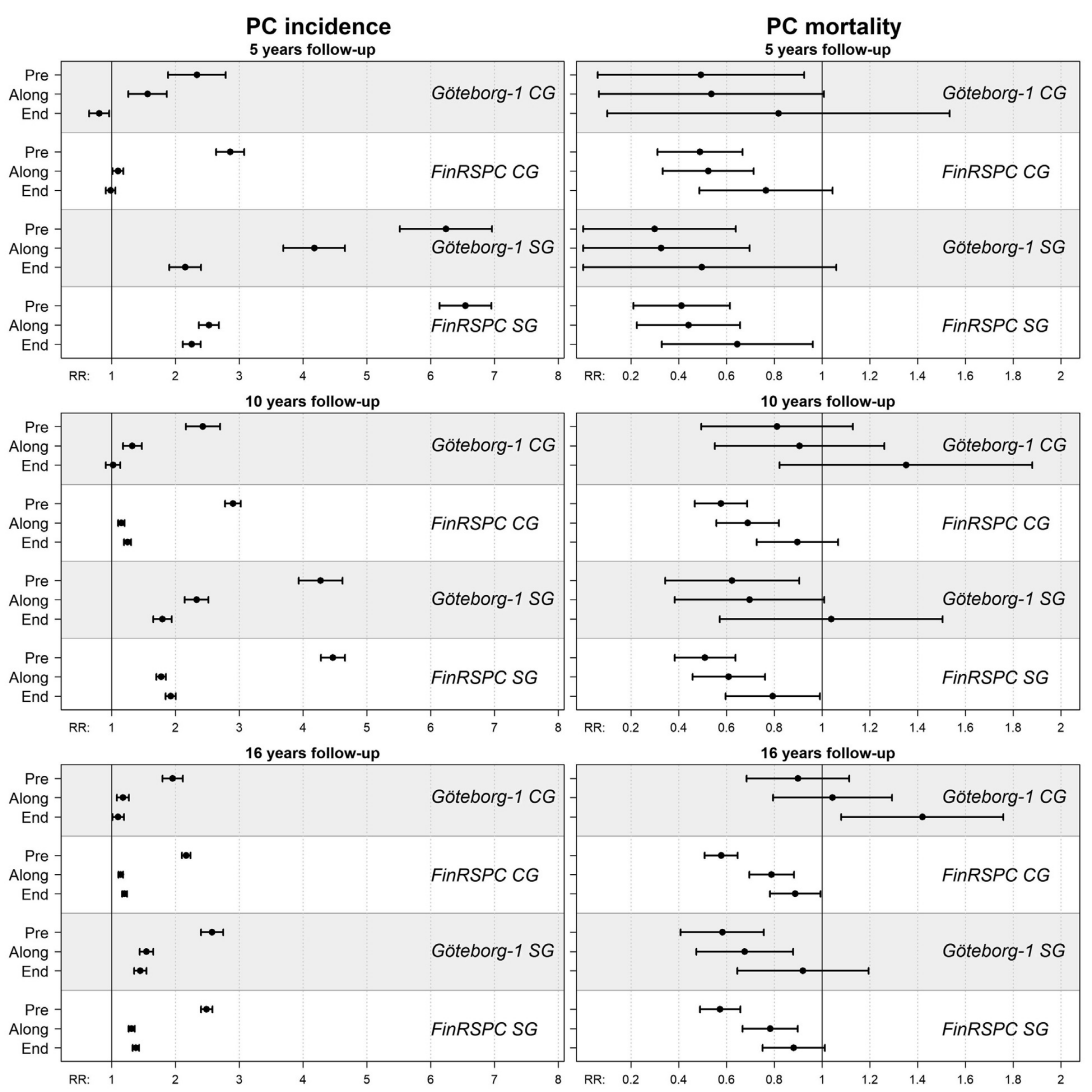
Ratios of the cumulative PC incidence and mortality in the CGs and SGs vs the expected rates based on pre-, along-, and end-study rates after 5, 10, and 16 year follow-up, with 95% confidence intervals. CG = control groups; PC = prostate cancer; SG = screening groups.

Cumulative incidence and mortality for trial data, and expected curves based on pre-study, along-study, and end-study incidence and mortality rates are presented in Figures 1A and 1B and Figures 2A and 2B (with CIs in Supplementary Fig. 1–2). Figure 3 and Supplementary Tables 3–4 show observed-to-expected ratios after 5, 10, and 16 year. In FinRSPC, the incidence in the CG was similar to that expected with end-study rates during the first 5 year, and 20% (95% CI: 17–24) higher at 16 year. In Göteborg-1, the observed incidence in the CG was lower than that expected with end-study rates for up to approximately 10 year, and at 16 year it was 10% (95% CI: 1–19) higher. The incidence in both SGs was higher than that expected with end-study rates at 16 year of follow-up.

In the SGs, the observed PC mortality after 16 year was 43% (95% CI: 34–51) lower than with pre-study rates in FinRSPC and 42% (95% CI: 24–59) in Göteborg-1. Compared with end-study rates, it was 12% (95% CI: -1–25) lower in FinRSPC and 8% (95% CI: -19–36) lower in Göteborg-1. For the Finnish CGs, the observed mortality was 42% (95% CI: 35–49) lower than with pre-study rates and 11% (95% CI: 1–22) lower than with end-study rates. For the Swedish CG, mortality was 10% (95% CI: -11–32) lower and 42% (95% CI: 8–76) higher, respectively.

## 4 Discussion

This study shows that PC incidence increased and mortality decreased from the pre-study period (1990–1994) to the end-study period (2010–2014) in a similar way in the Finnish and Swedish populations. Expected PC incidence and mortality were higher in Sweden than in Finland both for pre- and end-study rates. The excess PC incidence in the SG compared with the CG was higher in Göteborg-1 (44%) than in FinRSPC (21%), and the reduction in PC mortality was much larger in Göteborg-1 (36%) than in FinRSPC (2%), when age at randomization was restricted to 55–63 year.

When comparing PC mortality in the SGs in FinRSPC and Göteborg-1 with population rates, mortality was approximately 40% lower than expected based on pre-study rates, close to end-study levels, in both trials. However, the mortality in the Swedish CG was close to that expected based on pre-study levels, while in the Finnish CG mortality was close to that expected with end-study rates. This suggests that the small screening effect in FinRSPC is mainly due to an unexpectedly low PC mortality in the CG.

Similar mortality in the Swedish and Finnish SGs and a clear difference limited to the CGs were already shown in the 12 year follow-up of FinRSPC [8]. A proposed explanation for this difference observed is the high level of contamination of the Finnish CG [5], [11].

The PSA uptake in the Finnish population started around 1990, approximately 7 year earlier than in Sweden (See Fig. 1 in [12]). Before 1990, PC incidence was about 35% higher in Sweden, reflecting a higher baseline risk. Between 1995 and 2005 the incidence rates were similar, suggesting more extensive PSA testing in Finland than in Sweden given its lower baseline risk. FinRSPC started in 1996–1999, when PSA testing was already common in Finland. The incidence in the CG of FinRSPC increased rapidly and resembled the population rates along-study and at end-study, exceeding those after 7 year. Göteborg-1 started in 1995, and the PC incidence in the CG during the initial year of the trial was low, close to the pre-study levels (Fig. 1). However, over time contamination increased and after 12 year approximately 55% had undergone a PSA test [13]. In FinRSPC corresponding proportion was 63%, excluding private-sector testing (∼25% of primary care) [11]. Moreover, only 28% of elevated PSA tests led to a biopsy in the CG in Göteborg-1 and the corresponding figure in FinRSPC is unknown.

As approximately 85% of men dying from PC in the CGs were diagnosed with high-risk/advanced PC in both studies, it is obvious that a stage shift is a prerequisite for a major screening effect. The crucial role of stage shift for the screening effect in the ERSPC has been investigated [14], [15]. The detection of high-risk/advanced tumors in Göteborg-1 was much higher in the CG than in the SG, while in FinRSPC, the numbers were more similar between the groups (Supplementary Table 2). This suggests that a stage shift occurred in the Swedish SG, while either the stage shift in the SG was much smaller or the opportunistic testing in the CG also led to a stage shift in FinRSPC. The latter is supported by the early increase in incidence in the Finnish CG. The more intense screening (differences in PSA threshold and screening interval) in Göteborg-1 SG may also have resulted in a larger stage shift than in FinRSPC; however, previous studies consider this unlikely to explain the large difference in mortality reduction [16]. If the extent and timing of opportunistic testing in the CG substantially affect PC mortality, as suggested here, it is difficult to evaluate how protocol differences may have influenced the efficacy.

During the early year of the trials, opportunistic PSA testing was becoming widespread, but also new treatments were introduced. Surgery became more frequent in men with localized disease, and treatment of locally advanced PC with radiation and hormones together was shown to be more efficient than hormonal treatment alone [17]. Over the last 30 year, PC mortality has declined considerably in Western Europe. In Finland and Sweden, PC mortality in men younger than 80 year has declined by approximately 50% [18]. The decline in PC mortality started about 7 year after the rise in incidence, around 1997 in Finland, but 7–8 year later in Sweden. It was concluded that the rapid decline in PC mortality from the mid-1990s was consistent with a combined effect of PSA testing and improved treatments, although their relative contributions could not be disentangled [12].

In summary, the early rise of PSA testing and PC incidence and decline in PC mortality in the Finnish population may have reduced the potential for observing an effect of screening in the Finnish study. In combination with that men in FinRSPC were included on average 2.5 year later than men in Göteborg-1, this suggests that earlier and more extensive opportunistic testing in the FinRSPC CG, leading to a stage shift, had a major contribution to the difference in PC mortality reduction in the two studies.

The small PC mortality reduction in FinRSPC has implications for the future. Two other large studies, PLCO and the French ERSPC center, also had a high rate of contamination and did not show reductions in PC mortality [19], [20]. These results raise the question of whether ongoing randomized screening studies will be able to demonstrate conclusive evidence of reduction in PC mortality. Due to lower population PC mortality, demonstrating further reduction is more challenging compared to studies conducted around 30 year ago. Yet, a recent study found no clear relation between incidence and mortality reduction among European countries [21].

Strengths of the present study are that both Göteborg-1 and FinRSPC are population-based, used similar enrolment and randomization procedures, and have long follow-up of large cohorts in the same age span. Furthermore, Finland and Sweden have reliable population and cancer registers. The main limitation is the relatively few PC deaths in Göteborg-1, when stratified by tumor stage, leading to uncertainty in the estimated proportions. The expected incidence curves were based on national data, while the studies were performed mainly in metropolitan areas; however, regional and national statistics differ very little. The study is limited to men aged 55–63 year at randomization. Thus, the age groups with the strongest screening effects were excluded; 50–54 year in Göteborg-1 [22] and 67 year in FinRSPC [23]. In FinRSPC, controls aged 67 year had high PC incidence from study start, indicating exposure to extensive opportunistic testing (data not shown). A mortality benefit was still observed, suggesting opportunistic testing does not fully explain mortality differences. Finally, the study is exploratory, hence the findings are suggestive rather than confirmatory.

## 5 Conclusion

The lower PC mortality reduction in FinRSPC compared with Göteborg-1 is likely due to unexpectedly low mortality in the CG, related to the earlier uptake of PSA testing in Finland and the later start of FinRSPC, leading to higher PC incidence with an associated reduced PC mortality. This indicates difficulties in demonstrating a mortality benefit in new screening trials.

  ***Author contributions***: Marianne Månsson had full access to all the data in the study and takes responsibility for the integrity of the data and the accuracy of the data analysis.

  *Study concept and design*: Månsson, Hugosson.

*Acquisition of data*: Månsson, Talala, Auvinen, Hugosson.

*Analysis and interpretation of data*: Månsson, Talala, Auvinen, Hugosson.

*Drafting of the manuscript*: Månsson, Hugosson.

*Critical revision of the manuscript for important intellectual content*: Månsson, Talala, Auvinen, Hugosson.

*Statistical analysis*: Månsson.

*Obtaining funding*: Auvinen, Hugosson.

*Administrative, technical, or material support*: None.

*Supervision*: None.

*Other* (specify): None.

  ***Financial disclosures:*** Marianne Månsson certifies that all conflicts of interest, including specific financial interests and relationships and affiliations relevant to the subject matter or materials discussed in the manuscript (eg, employment/affiliation, grants or funding, consultancies, honoraria, stock ownership or options, expert testimony, royalties, or patents filed, received, or pending), are the following: Professor Anssi Auvinen has received lecture fees from IPSEN. All other authors report no conflict of interest.

  ***Funding/Support and role of the sponsor*:** Professor Anssi Auvinen was supported by Research Council of Finland (Grant No. 260931), Cancer Foundation Finland and State Research Funding administered by Tampere University Hospital. Professor Jonas Hugosson was supported by the Swedish Cancer Society, grants from the Swedish state under the agreement between the Swedish government and the county councils, the ALF-agreement (number 966044), the Swedish Research Council, Biocare, Regional Cancer Center Western Region Sweden, and the Swedish Prostate Cancer Association.

  ***Acknowledgments:*** We thank all the members of the Göteborg-1 and FinRSPC trials, in particular data manager Helén Ahlgren and study nurse Maria Nyberg in Göteborg-1 and Teuvo Tammela, Kimmo Taari, and Paula Kujala in FinRSPC.

  ***Declaration of generative AI and AI-assisted technologies in the manuscript preparation process***: During the preparation of this work, the authors used ChatGPT (OpenAI, San Francisco, CA, USA) to improve grammar and language clarity. After using this tool, the authors reviewed and edited the content as needed and take full responsibility for the content of the published article.

## Appendix A Supplementary data

Supplementary Data 1 Supplementary graphs and tables.
