# Supplementary material for: Comparison of the Swedish and Finnish Centers in the European Randomized Study of Screening for Prostate Cancer (ERSPC)
Source: Eur Urol Open Sci. 2026 May 15;88:107–15. doi: 10.1016/j.euros.2026.04.008 (PMC13202249; doi:10.1016/j.euros.2026.04.008)
Supplement: Supplementary Data 1 — Supplementary graphs and tables. [file mmc1.docx]

# Supplementary Materials

Supplement to: Månsson, Talala, Auvinen, Hugosson –

Comparison of the Swedish and Finnish Centers in the European Randomized Study of Screening for Prostate Cancer (ERSPC)

Supplementary Figures 1 and 2 are identical to Figures 1 and 2, but here the curves based on observed study data are provided with 95% confidence intervals.

**Supplementary Figure 1**

Observed cumulative incidence of PC in the control and screening groups of FinRSPC (A) and Göteborg-1 (B), and estimations of the expected incidence based on population rates from pre-study (1989-1994), along-study and end-study (2010-2014)


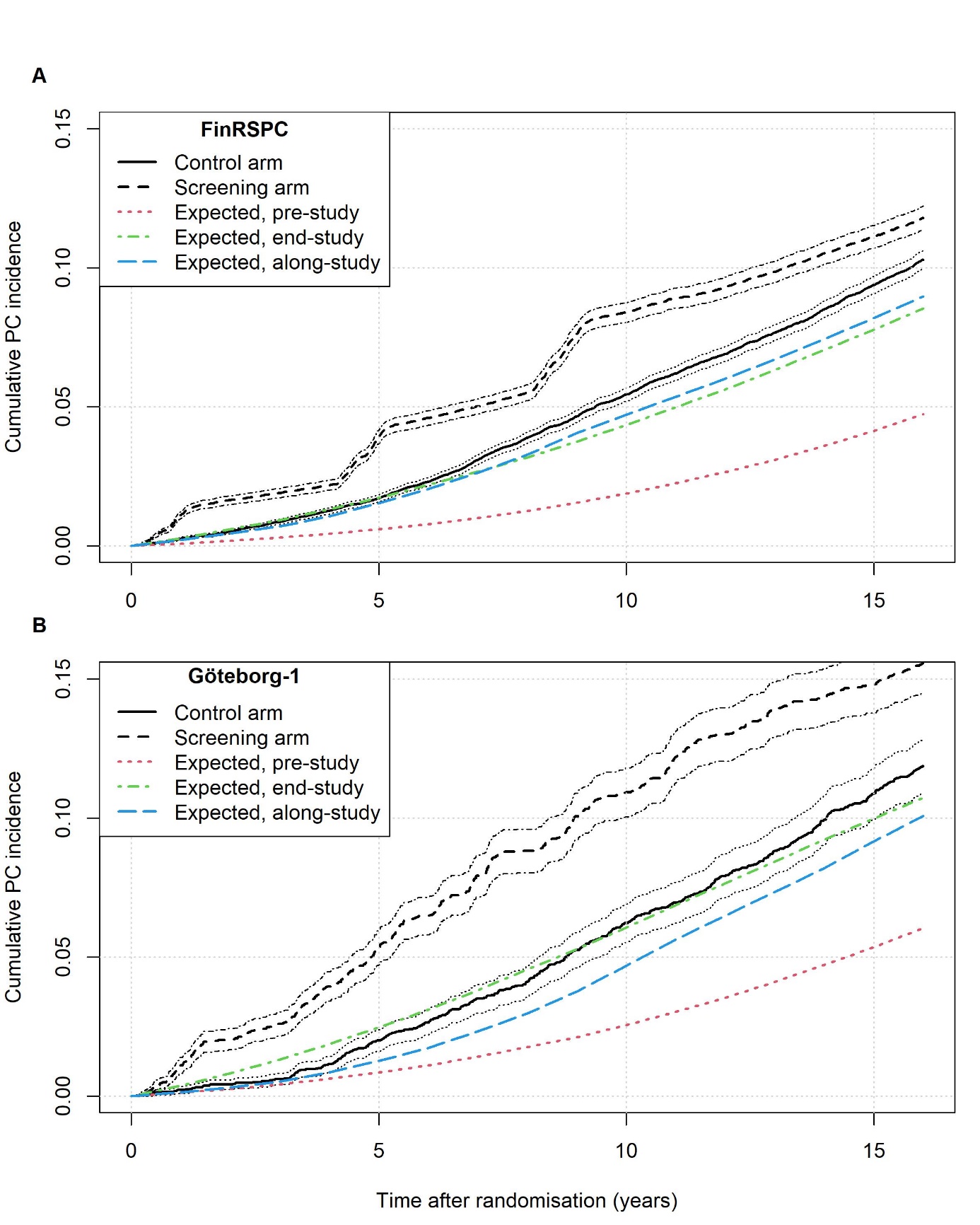


**Supplementary Figure 2**

Observed cumulative PC mortality in the control and screening groups of FinRSPC (A) and Göteborg-1 (B), and estimations of the expected mortality based on population rates from pre-study (1989-1994), along-study and end-study (2010-2014)


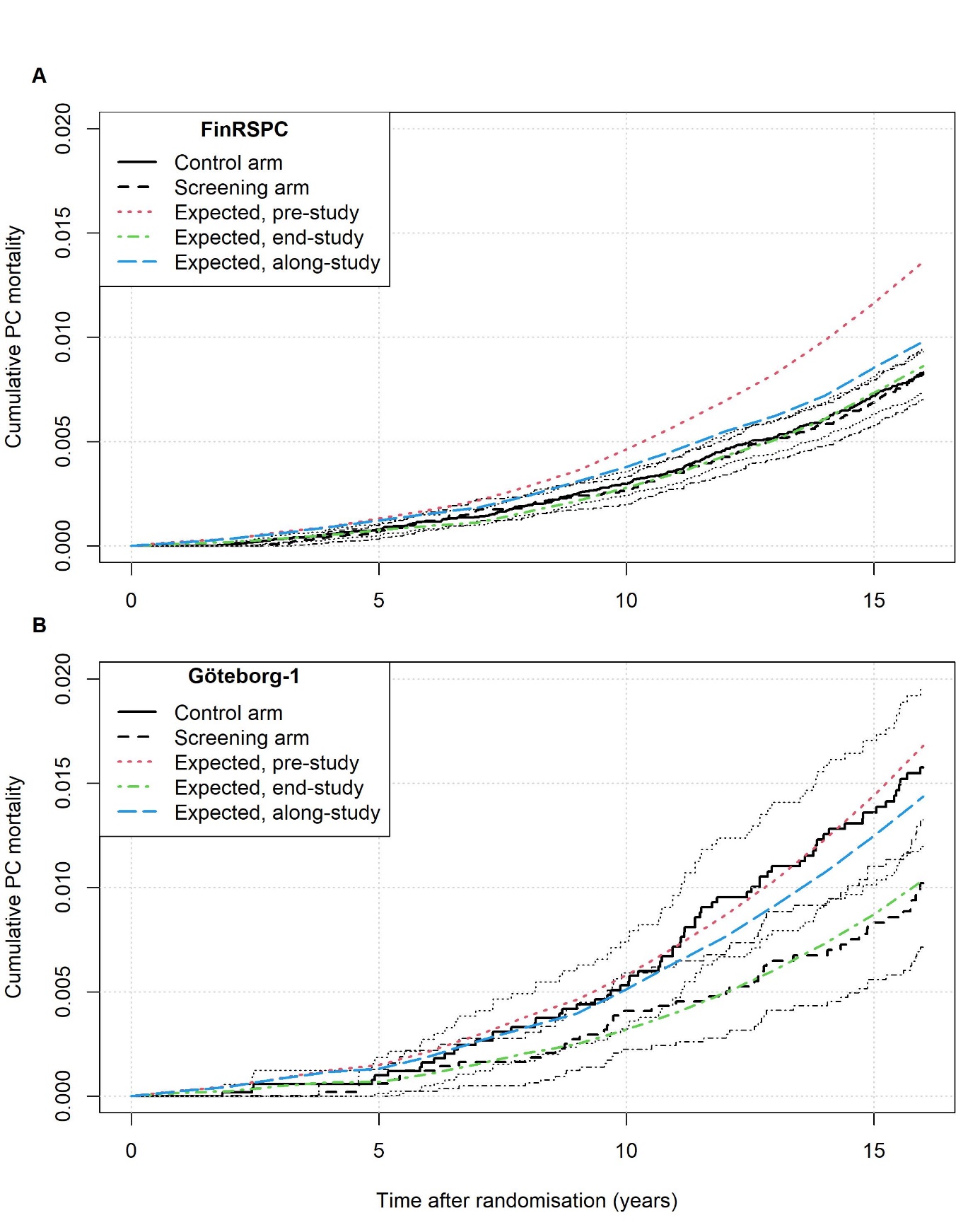


**Supplementary Table 1**

Details of the screening algorithms in Göteborg-1 and FinRSPC

|  | **Göteborg-1** | **FinRSPC** |
| --- | --- | --- |
| Period of randomization | 31 December 1994 | 1 January 1996 to 1999 |
| Type of randomization | Before consent | Before consent |
| Ages at randomization | 50 to 64 | 55, 59, 63, or 67 |
| Screening interval | 2 years | 4 years |
| Upper age of screening | 67 to 71 years | 71 years |
| Number of screening rounds | 3 times (age 64 at randomization) up to  10 times (age 50 at randomization) | - 2 times (age 67 at randomization) - 3 times (age 55, 59 or 63 at randomization |
| Indication for biopsy | PSA ≥ 3ng/ml | - PSA ≥ 4.0ng/mL   OR   - PSA 3.0 - 3.99ng/mL AND - suspicious DRE (1996-1998) - free/total PSA ≤ 16% (1999 onwards) |
| Biopsy type | - Up to 2008: Sextant biopsy - 2009 onwards : 10 cores | - Up to 2001: Sextant biopsy - 2002 onwards : 10-12 cores |
| Cause of death | Cause-of-death committee for all men diagnosed with PC | - 1996-2003: Cause-of-death committee for all men diagnosed with PC.   There was an excellent agreement with the official causes-of-death register (Mäkinen 2008) leading to the use of official register.   - 2004 onwards: Official causes of death register |

^1^ Mäkinen T, Karhunen P, Aro J, Lahtela J, Määttänen L, Auvinen A. Assessment of causes of death in a prostate cancer screening trial. Int J Cancer. 2008;122(2):413-7.

**Supplementary Table 2**

Risk of PC during 16 years follow-up in the different tumor stage group among all men, both screen-detected and PC detected outside the study. Risk ratios between SG and CG for each stage group and center

Definitions: Low risk: cT1/cT2, GS 6 and PSA<10; Intermediate risk: (cT1/cT2, PSA 10-20, and GS = 6) or (cT1/cT2, PSA <20 and GS=7); High risk: (cT1/cT2, PSA≥20 and GS<=7) or (cT1/cT2 and GS=8-10) or (cT3); Advanced: N1, M1, cT4 or PSA >100.

| Tumor stage group | FinRSPC CG  n men = 39,035 | FinRSPC SG  n men = 25,739 | FinRSPC  Risk ratio, SG / CG  (95% CI) | Göteborg-1 CG  n men = 5,357 | Göteborg-1 SG  n men = 5,346 | Göteborg-1  Risk ratio, SG / CG  (95% CI) |
| --- | --- | --- | --- | --- | --- | --- |
|  | n (%) | n (%) |  | n (%) | n (%) |  |
| Low | 955 (2.4) | 1169 (4.5) | 1.86 (1.71, 2.02) | 163 (3) | 434 (8.1) | 2.67 (2.24, 3.18) |
| Moderate | 1144 (2.9) | 716 (2.8) | 0.95 (0.87, 1.04) | 158 (2.9) | 169 (3.2) | 1.07 (0.87, 1.33) |
| High | 843 (2.2) | 526 (2) | 0.95 (0.85, 1.05) | 106 (2) | 71 (1.3) | 0.67 (0.50, 0.90) |
| Advanced | 368 (0.9) | 162 (0.6) | 0.67 (0.56, 0.80) | 78 (1.5) | 44 (0.8) | 0.57 (0.39, 0.81) |
| Not known | 152 (0.4) | 121 (0.5) | 1.21 (0.95, 1.53) | 16 (0.3) | 5 (0.1) | 0.31 (0.12, 0.82) |
| No cancer | 35573 (91) | 23045 (90) | 0.98 (0.98, 0.99) | 4836 (90) | 4623 (86) | 0.96 (0.94, 0.97) |

**Supplementary Table 3**

Cumulative incidence of prostate cancer after 5-, 10- and 16-years follow-up based on study data and register data, and relative rates of incidence between observed and expected (register data) incidences with 95% confidence intervals

|  | **5 years** | **10 years** | **16 years** |
| --- | --- | --- | --- |
| **FinRSPC** |  |  |  |
| Control group | 1.7 (1.6, 1.8) | 5.4 (5.2, 5.7) | 10.3 (10.0, 10.6) |
| Screening group | 3.9 (3.7, 4.1) | 8.4 (8.0, 8.7) | 11.8 (11.4, 12.2) |
| Expected pre-study (1989-1994) | 0.6 | 1.9 | 4.7 |
| Expected end-study (2010-2014) | 1.7 | 4.4 | 8.5 |
| Expected along-study | 1.5 | 4.7 | 9.0 |
| CG / pre-study | 2.86 (2.64, 3.08) | 2.90 (2.78, 3.03) | 2.17 (2.10, 2.24) |
| CG / end-study | 0.99 (0.91, 1.06) | 1.25 (1.20, 1.31) | 1.20 (1.17, 1.24) |
| CG / along-study | 1.10 (1.02, 1.19) | 1.16 (1.11, 1.21) | 1.15 (1.11, 1.18) |
| SG / pre-study | 6.55 (6.14, 6.95) | 4.47 (4.28, 4.66) | 2.49 (2.40, 2.58) |
| SG / end-study | 2.26 (2.12, 2.40) | 1.93 (1.85, 2.01) | 1.38 (1.33, 1.43) |
| SG / along-study | 2.53 (2.37, 2.68) | 1.78 (1.70, 1.85) | 1.32 (1.27, 1.36) |
| **Göteborg-1** |  |  |  |
| Control group | 2.0 (1.6, 2.4) | 6.2 (5.5, 6.9) | 11.9 (10.9, 12.8) |
| Screening group | 5.3 (4.7, 6.0) | 10.9 (10.0, 11.8) | 15.6 (14.5, 16.6) |
| Expected pre-study (1989-1994) | 0.9 | 2.6 | 6.1 |
| Expected end-study (2010-2014) | 2.5 | 6.1 | 10.7 |
| Expected along-study | 1.3 | 4.7 | 10.1 |
| CG / pre-study | 2.34 (1.89, 2.79) | 2.43 (2.16, 2.70) | 1.96 (1.80, 2.12) |
| CG / end-study | 0.81 (0.65, 0.96) | 1.03 (0.91, 1.14) | 1.10 (1.01, 1.19) |
| CG / along-study | 1.57 (1.26, 1.87) | 1.33 (1.18, 1.47) | 1.18 (1.08, 1.27) |
| SG / pre-study | 6.24 (5.52, 6.96) | 4.28 (3.93, 4.62) | 2.58 (2.40, 2.75) |
| SG / end-study | 2.15 (1.90, 2.40) | 1.80 (1.66, 1.95) | 1.45 (1.35, 1.55) |
| SG / along-study | 4.18 (3.69, 4.66) | 2.33 (2.14, 2.52) | 1.55 (1.44, 1.65) |

**Supplementary Table 4**

Cumulative mortality of prostate cancer after 5-, 10- and 16-years follow-up based on study data and register data, and relative rates of incidence between observed and expected (register data) incidences with 95% confidence intervals

|  | **5 years** | **10 years** | **16 years** |
| --- | --- | --- | --- |
| **FinRSPC** |  |  |  |
| Control group | 0.08 (0.05, 0.11) | 0.30 (0.24, 0.35) | 0.83 (0.73, 0.93) |
| Screening group | 0.07 (0.03, 0.10) | 0.26 (0.20, 0.33) | 0.82 (0.70, 0.94) |
| Expected pre-study (1989-1994) | 0.16 | 0.52 | 1.43 |
| Expected end-study (2010-2014) | 0.10 | 0.33 | 0.93 |
| Expected along-study | 0.15 | 0.43 | 1.05 |
| CG / pre-study | 0.49 (0.31, 0.67) | 0.58 (0.47, 0.69) | 0.58 (0.51, 0.65) |
| CG / end-study | 0.76 (0.49, 1.04) | 0.90 (0.73, 1.07) | 0.89 (0.78, 0.99) |
| CG / along-study | 0.52 (0.33, 0.71) | 0.69 (0.56, 0.82) | 0.79 (0.69, 0.88) |
| SG / pre-study | 0.41 (0.21, 0.61) | 0.51 (0.38, 0.64) | 0.57 (0.49, 0.66) |
| SG / end-study | 0.64 (0.33, 0.96) | 0.79 (0.60, 0.99) | 0.88 (0.75, 1.01) |
| SG / along-study | 0.44 (0.23, 0.66) | 0.61 (0.46, 0.76) | 0.78 (0.67, 0.90) |
| **Göteborg-1** |  |  |  |
| Control group | 0.10 (0.01, 0.19) | 0.53 (0.32, 0.74) | 1.58 (1.20, 1.95) |
| Screening group | 0.06 (0.00, 0.13) | 0.41 (0.22, 0.59) | 1.02 (0.72, 1.33) |
| Expected pre-study (1989-1994) | 0.20 | 0.65 | 1.75 |
| Expected end-study (2010-2014) | 0.12 | 0.39 | 1.11 |
| Expected along-study | 0.18 | 0.59 | 1.51 |
| CG / pre-study | 0.49 (0.06, 0.92) | 0.81 (0.49, 1.13) | 0.90 (0.68, 1.11) |
| CG / end-study | 0.82 (0.10, 1.53) | 1.35 (0.82, 1.88) | 1.42 (1.08, 1.76) |
| CG / along-study | 0.54 (0.07, 1.01) | 0.91 (0.55, 1.26) | 1.04 (0.79, 1.29) |
| SG / pre-study | 0.30 (0.00, 0.64) | 0.62 (0.34, 0.90) | 0.58 (0.41, 0.76) |
| SG / end-study | 0.50 (0.00, 1.06) | 1.04 (0.57, 1.50) | 0.92 (0.64, 1.19) |
| SG / along-study | 0.33 (0.00, 0.70) | 0.70 (0.38, 1.01) | 0.68 (0.47, 0.88) |
